# Clinical Significance of OX40 and OX40 Ligand in the Peripheral Blood of Patients with Myasthenia Gravis

**DOI:** 10.1155/2022/4337399

**Published:** 2022-02-28

**Authors:** Xiaoling Zhou, Xiaoyuan Wang, Yanzheng Gu, Lan Chen, Yueping Shen, Jingluan Tian, Chen Shujun, Mingyuan Wang, Xiaoyu Duan, Hanqing Gao, Xiaopei Ji, Qi Fang, Xueguang Zhang, Qun Xue

**Affiliations:** ^1^Department of Neurology, First Affiliated Hospital of Soochow University, Suzhou, Jiangsu 215006, China; ^2^Medical College of Soochow University, Suzhou, Jiangsu 215006, China; ^3^Jiangsu Institute of Clinical Immunology, Jiangsu Key Laboratory of Clinical Immunology, First Affiliated Hospital of Soochow University, Suzhou, Jiangsu 215006, China; ^4^Department of Neurology, Nantong First People's Hospital, Nantong, Jiangsu 226000, China; ^5^Department of Epidemiology and Health Statistics, Soochow University, Suzhou, Jiangsu 215006, China; ^6^Suzhou Red Cross Central Blood Station, Suzhou, Jiangsu 215006, China

## Abstract

**Background:**

A previous study on thymomas in myasthenia gravis (MG) patients indicated that OX40 expression may be upregulated in thymic tissues adjacent to germinal centers (GCs) and thymomas, and OX40 may interact with OX40L in GCs to enhance anti-acetylcholine receptor antibody production. However, little is known about the clinical significance of the expression of OX40 and OX40L in the peripheral blood of patients with MG. We aimed to characterize the expression of membrane-bound and soluble OX40 and OX40L in the peripheral blood of patients with MG and to identify their clinical significance.

**Methods:**

For membrane molecules, we collected peripheral blood (PB) from 39 MG patients at baseline, 22 patients in relapse, and 42 patients in remission, as well as from 36 healthy participants as controls. For soluble molecules, plasma from 37 MG patients at baseline, 34 patients in relapse, and 30 patients in remission, as well as plasma from 36 healthy controls (HC), was retrospectively collected from the sample bank of the First Hospital of Soochow University. The expression of membrane-bound OX40 and OX40L (mOX40 and mOX40L) by immune cells was measured using flow cytometry. Plasma levels of soluble OX40 and OX40L (sOX40 and sOX40L) were measured by ELISA.

**Results:**

(1) The expression of OX40 on CD4+ T cells and that of OX40L on B cells and monocytes were significantly increased, and the levels of sOX40 were significantly decreased in MG patients at baseline compared with HC, while the expression of sOX40L was not significantly different between the two groups. (2) Dynamic observation of the molecules showed significantly higher expression of OX40 on CD4^+^ T cells and higher levels of sOX40 in MG patients in relapse than in MG patients at baseline and MG patients in remission. Furthermore, the expression levels of sOX40 were significantly elevated in MG patients in remission compared with MG patients at baseline, and the expression of sOX40L was significantly lower in MG patients in remission than in MG patients at baseline and MG patients in relapse. (3) Plasma levels of sOX40 and sOX40L were significantly decreased in 13 patients with relapsed MG after immunosuppressive treatment compared with those before treatment. (4) Correlation analysis showed that the expression of OX40 on CD4^+^ T cells in patients with relapsed MG was positively correlated with the concentration of acetylcholine receptor antibodies (AchR-Ab), whereas the expression of OX40L on CD19^+^ B cells and CD14^+^ monocytes was negatively correlated with disease duration. (5) Binary regression analysis showed that patients with high CD4^+^ OX40 expression and high sOX40L levels had an increased risk of relapse.

**Conclusions:**

OX40 and OX40L are abnormally expressed in the peripheral blood of patients with MG and may be closely associated with disease status and treatment. The OX40/OX40L pathway may be involved in the immunopathological process of MG and may play a role mainly in the later stage of MG.

## 1. Introduction

Myasthenia gravis (MG) is an antibody-mediated autoimmune disease of the neuromuscular junction. The main clinical manifestations of MG are fluctuating weakness and fatigue of the affected skeletal muscles [1]. MG is considered to be a classical humoral immune disease, and autoantibody production at the neuromuscular junction is the main pathological mechanism. However, recent studies have shown that T cells are engaged in the production of pathological antibodies in MG [2–4], suggesting that T-B cell interactions play an essential role in the pathogenesis of MG.

T cell activation requires a first signal provided by the major histocompatibility complex- (MHC-) peptide complex and a second signal delivered by costimulatory molecules. A lack of costimulatory signals can lead to the inability of T cells to respond and even programmed cell death [5]. OX40 (also named CD134, TNFRSF4, or ACT35) and its cognate ligand, OX40L (also called CD252, TNFSF4, gp34, or CD134L), are members of the tumor necrosis factor receptor (TNFR) and the tumor necrosis factor (TNF) superfamilies, respectively, and these molecules play important roles in regulating the immune response. OX40 is a type I transmembrane glycoprotein that is mainly expressed by activated CD4^+^ T cells. OX40L is a type II transmembrane glycoprotein that is predominantly expressed by antigen-presenting cells (ADCs), such as B cells, dendritic cells (DCs), and macrophages [6, 7]. OX40-OX40L interactions promote T cell proliferation, differentiation, memory, and survival, increase effector cytokine secretion, and suppress regulatory T cell function [8]. The OX40/OX40L pathway plays a significant role in the pathogenesis of human autoimmune diseases, including multiple sclerosis (MS) [9], systemic lupus erythematosus (SLE) [10], rheumatoid arthritis (RA) [11], and type 1 diabetes [12], and the expression of OX40 on CD4^+^ T cells correlates with disease severity in patients with SLE [13, 14]. Blockade of OX40-OX40L interactions ameliorates disease in many animal models of autoimmunity [15].

In addition to their expression in membrane-bound forms on peripheral circulating lymphocytes, OX40 and OX40L are also expressed in soluble forms in plasma [8]. Soluble molecules regulate the OX40/OX40L axis by binding to corresponding membrane molecules and increase the diversity and complexity of the OX40/OX40L pathway. There are few studies on the expression and clinical value of OX40 and OX40L in MG. A previous study on thymomas in MG patients indicated that OX40 expression in a fraction of activated CD4^+^ T cells may be upregulated in thymic tissues that are adjacent to germinal centers (GCs) and thymomas in MG, and OX40 may interact with OX40L in GCs to enhance anti-acetylcholine receptor antibody production in MG [16]. However, little is known about the clinical significance of the expression of OX40 and OX40L in the peripheral blood of MG patients, and few studies have been carried out on the soluble forms of these molecules. Whether OX40 and OX40L (both the membrane and soluble forms) are aberrantly expressed in the peripheral blood of patients with MG and whether such aberrant changes are associated with the onset, progression, remission, relapse, and disease severity of MG are worth investigating. The objectives of this study were to characterize the expression of OX40 and OX40L in the peripheral blood of patients with different stages of MG, to analyze the correlation of OX40 and OX40L levels with clinical indicators, and to observe the effects of immunosuppressive drugs (including glucocorticoids and immunosuppressants) on the soluble forms of the molecules.

## 2. Materials and Methods

### 2.1. Patients and Controls

This study was approved by the Ethics Committee of Soochow University, China. All the study participants signed informed consent forms. Between July 2019 and June 2021, patients with MG at baseline, patients with MG in remission, and patients with MG in relapse were recruited from the Department of Neurology of the First Affiliated Hospital of Soochow University. All the patients were diagnosed with MG based on the presence of typical clinical manifestations together with one or more of the following criteria: (a) seropositivity for acetylcholine receptor antibodies (AchR-Abs), (b) decreased amplitude of compound muscle action potentials by more than 10% in response to repetitive low-frequency nerve stimulation, (c) positive response to fatigue test, and (d) positive reaction to neostigmine.

The inclusion criteria were as follows: (a) patients with MG in the baseline, relapse, and remission stages, (b) patients of both sexes with age ≥ 18 years, and (c) patients who voluntarily signed the informed consent forms. The exclusion criteria were as follows: (a) patients with other autoimmune diseases, (b) patients with malignancies (except thymoma), (c) patients with severe infections (both acute and chronic infectious diseases), and (d) patients with dementia or mental disorders or who were pregnant.

Simultaneously, sex-, age-, and race-matched healthy controls (HCs) were recruited from the hospital's physical examination center.

### 2.2. Diagnosis of Baseline, Remission, and Relapse

Baseline MG was defined as MG at the initial onset that had not yet been treated with immunosuppressive therapy, plasma-exchange therapy, or intravenous immunoglobulins. According to the 2016 International Consensus Guidance for the Management of Myasthenia Gravis [17], remission of MG was defined as a lack of symptoms or functional limitations associated with MG but the presence of some weakness upon examination of certain muscles. To date, there have been no uniform criteria for the diagnosis of relapse of MG. In our study, patients with relapse of MG were defined as follows: (a) patients who had previously received effective treatment and reached their treatment goals, that is, minimal manifestation status (MMS) or better as classified by the Myasthenia Gravis Foundation of America (MGFA) [18] Task Force postintervention status (PIS) [19]; (b) patients who experienced the reappearance of any signs or symptoms of muscle weakness, for whom the recurrence of signs and symptoms lasted more than 24 hours and for whom the period between the relapse of MG and last remission was at least 30 days [20]; and (c) patients whose quantitative MG score (QMGs) increased by ≥3 points compared to that measured during remission [21].

### 2.3. Treatment Strategies

All the patients received regular treatment after admission to the hospital. Cholinesterase inhibitors were used for symptomatic treatment. Patients with MG received regular doses of pyridostigmine (180-240 mg/d) at the onset of the disease, and the doses of pyridostigmine were adjusted as needed according to symptoms. Patients with generalized MG (GMG) in the acute exacerbation phase received induction treatments of intravenous immunoglobulin (IVIG, 400 mg/kg/d ∗5 d) or plasma exchange to achieve MMS, which was maintained by immunosuppressive treatments (including corticosteroids and/or immunosuppressants) to prevent relapse.

Corticosteroids or immunosuppressants were used in patients with MG who did not achieve their treatment goals after an adequate pyridostigmin trial. There were 2 protocols for the use of glucocorticosteroids at the disease onset to achieve MMS. Patients received high doses of steroids (1-1.5 mg/kg/d) that were maintained for 1-2 months to achieve improvements in symptoms. Other patients were treated with gradually increasing steroid doses, starting with a dose of 20 mg/d and doses increasing by 10 mg per week until reaching the target dose (0.5-1.0 mg/kg/d); then, this dose was maintained for 6-8 weeks after the primary symptoms improved. Then, the steroids began to be reduced by 5-10 mg every 2-4 weeks and by 5 mg every 4 to 8 weeks until reaching a dose of 20 mg.

To decrease glucocorticosteroid use, immunosuppressants were added before MMS until a stable dose was achieved. Stable doses of various immunosuppressants were as follows: 2-3 mg/kg/d azathioprine, 0.05-0.1 mg/kg/d tacrolimus, 1-3 g/d mycophenolate mofetil, and 400-800 mg/week cyclophosphamide intravenously. Combined regimens were defined as a combination of low-dose steroids and stable doses of immunosuppressants. Low doses of steroids were defined as <0.25 mg/kg/d prednisone or methylprednisolone [22].

### 2.4. Collection of Clinical Data

The primary clinical data of the patients were collected, including time when the patient was first diagnosed with MG, time of remission, time of recurrence, time of follow-up, demographic data (sex and age at onset), disease duration, MGFA classification, thymic pathology, serum anti-AchR antibody titers, MGFA Task Force postintervention status, treatment (no treatment or cholinesterase inhibitor treatment and immunosuppressive therapy), and QMGs. Disease duration was defined as the time from the first appearance of a patient's symptoms of muscle weakness to the time when the patient came to the hospital [23].

### 2.5. Sample Processing

Peripheral blood was collected from patients at baseline, in relapse, and in remission and from healthy controls. In the morning, 4 ml of peripheral fasting venous blood was collected from all the subjects and placed into 2 EDTA anticoagulation tubes. One tube was utilized for immunofluorescence labeling and flow cytometry analysis. One tube was centrifuged at 3000 rpm for 20 minutes, after which the top-layer plasma sample was collected and frozen at -80°C for future usage.

### 2.6. Flow Cytometry

Fifty microliters of peripheral blood was taken from each subject's EDTA anticoagulation tube, and the following fluorescently labeled monoclonal antibodies were used for staining: FITC-conjugated anti-human CD19, FITC-conjugated anti-human 14, FITC-conjugated anti-human OX40, PE-conjugated anti-human OX40L, and PC5-conjugated anti-human CD4 (all antibodies were purchased from BioLegend, San Diego, CA, USA). Then, the cells were incubated for 30 minutes at room temperature in the dark. Next, we added 200 *μ*l of red blood cell lysis buffer (purchased from Beckman Coulter, Brea, CA, USA) and incubated the mixtures for 20 minutes at 37°C. Finally, every specimen was washed with 1 ml of PBS, centrifuged at 2000 rpm for 5 minutes, resuspended in 500 *μ*l of PBS, and assessed using a flow cytometer (Beckman Coulter, Brea, CA, USA). FlowJo version 10.4 software was used to analyze the primary flow cytometry data.

### 2.7. ELISAs

Frozen plasma specimens were thawed at room temperature and then centrifuged at 2000 rpm for 5 minutes. The plasma supernatant was collected for enzyme-linked immunosorbent assays (ELISAs). Human acetylcholine receptor autoantibody ELISA kits (procured from RSR Biotechnology, UK) were used to detect the concentration of AchR-Abs. Plasma levels of sOX40 and sOX40L were measured with Human sOX40 and sOX40L kits (purchased from Shanghai Kanglang Biotechnology Co., Ltd., China). The specific experimental procedures for each ELISA were carried out with strict adherence to the manufacturer's instructions.

### 2.8. Statistical Analyses

SPSS version 26.0 and GraphPad Prism 8.0 software were used for statistical analyses. Quantitative data with a normal distribution are reported as the means and standard deviations, quantitative data with a nonnormal distribution are presented as medians and interquartile ranges, and qualitative data are expressed as frequencies and percentages. Two independent samples were compared using *t*-tests (normal distribution) or Mann–Whitney *U* tests (nonnormal distribution). Comparisons between two paired samples were made by the paired *t*-test. Multiple independent samples were compared using one-way ANOVA (normal distribution) or Kruskal–Wallis *H* test (nonnormal distribution), and the Mann–Whitney *U* test with the Bonferroni correction was used for comparisons between groups. The chi-square test and Fisher's exact test were utilized for the comparison of qualitative variables. Nonparametric Spearman correlation was used for the analysis of correlations between two continuous variables that did not conform to a normal distribution. A univariate logistic regression model was used to analyze the factors that affect recurrence in patients with MG, and factors with *P* values < 0.1 in the univariate regression analysis were further included in the multivariate logistic regression model to identify the independent risk factors that affect recurrence of MG. A *P* value < 0.05 was regarded as statistically significant.

## 3. Results

### 3.1. Study Population

The demographic and clinical characteristics of all the study participants are summarized in Table 1. For membrane molecules, between July 2019 and June 2021, we recruited 39 patients with MG at baseline, 22 patients with MG in relapse, and 42 patients with MG in remission, along with 36 healthy individuals as controls. Demographic characteristics (both age and sex) were comparable between the four groups. The proportion of thymoma patients with MG at baseline was 17.9%, those with MG in remission were 16.7%, and those with MG in relapse were 40.9%. The three groups (baseline, remission, and relapse) were comparable in terms of the proportion of MG patients with thymomas, the proportion of patients with early-onset MG (EOMG), and the proportion of patients with late-onset MG (LOMG) (*P* > 0.05), but there were some differences among the 3 groups regarding MGFA classification (*P* ≤ 0.001), AchR-Ab positivity rate (*P* = 0.003), disease duration (*P* ≤ 0.001), and QMGs (*P* ≤ 0.001).

For soluble molecules, plasma from 37 patients with MG at baseline, 34 patients in relapse, and 30 patients in remission, as well as plasma from 36 healthy controls, was retrospectively collected from July 2017 to May 2021 from the sample bank of the First Hospital of Soochow University. There were no significant differences among the 4 groups in terms of sex or age (*P* > 0.05). The rate of AchR-Ab positivity was 86.4% for MG patients at baseline, 100% for MG patients in remission, and 100% for MG patients in relapse. The three groups were comparable regarding the rate of AchR-Ab positivity and the proportion of patients with EOMG and LOMG (*P* > 0.05). However, there were some differences in terms of the MGFA classification (*P* ≤ 0.001), the proportion of patients with thymoma (*P* = 0.005), disease duration (*P* ≤ 0.001), and QMGs (*P* ≤ 0.001) among the three groups.

### 3.2. mOX40 and mOX40L Were Highly Expressed on Peripheral Blood Lymphocytes from Patients with MG at Baseline

**Figure 1:**
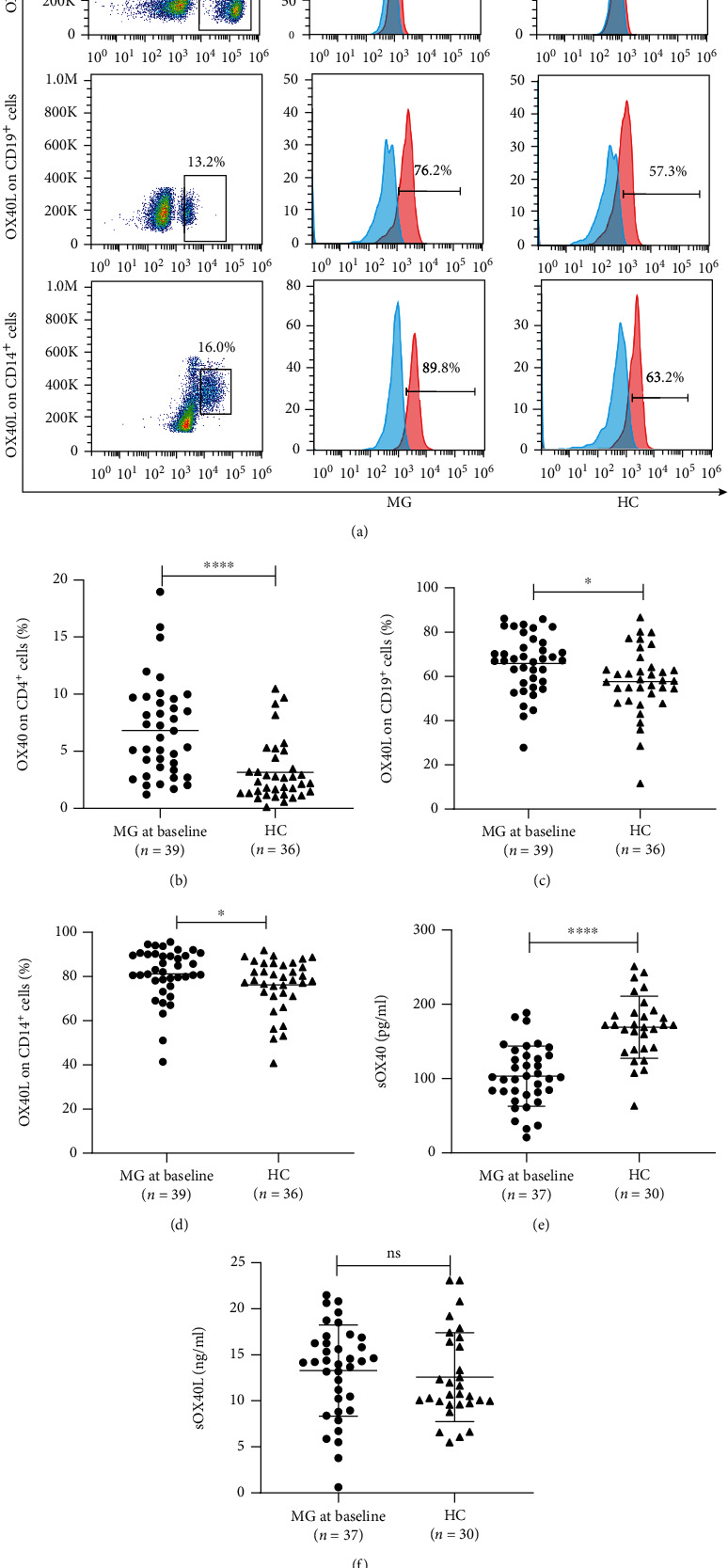
OX40/OX40L expression on the peripheral blood lymphocytes and plasma of patients with MG at baseline. (a) Representative expression of OX40 on CD4^+^ T cells and OX40L on CD19^+^ B cells and CD14^+^ mononuclear cells in the patients with MG at baseline and HC groups. The red lines indicate specific staining results measured by flow cytometry, and the blue lines indicate isotype controls. (b) Comparison of OX40 expression on CD4^+^ T cells in the patients with MG at baseline and HC groups. (c) Comparison of OX40L expression on CD19^+^ B cells in the patients with MG at baseline and HC groups. (d) Comparison of OX40L expression on CD14^+^ mononuclear cells in the patients with MG at baseline and HC groups. (e) Comparison of sOX40L plasma levels in the patients with MG at baseline and HC groups. (f) Comparison of sOX40L plasma levels in the patients with MG at baseline and HC groups.

Flow cytometry was used to analyze the expression of OX40 and OX40L on peripheral blood lymphocytes in patients with MG at baseline (*n* = 39) and in healthy subjects (*n* = 36) (Table 2). The expression of OX40 on CD4^+^ T lymphocytes was increased (*P* ≤ 0.001) (Figures 1(a) and 1(b)), and the expression of OX40L on CD19^+^ B cells and CD14^+^ monocytes was increased in the peripheral blood of MG patients at baseline compared with that of HC (*P* = 0.011 and *P* = 0.026, respectively) (Figures 1(a), 1(c), and 1(d)).

### 3.3. Plasma Levels of sOX40, but Not of sOX40L, Were Decreased in Patients with MG at Baseline

Plasma levels of sOX40 and sOX40L in patients with MG at baseline (*n* = 37) and in healthy volunteers (*n* = 30) were measured by ELISA (Table 2). The sOX40 levels in the patients with MG at baseline were significantly reduced compared with those in the HC (*P* ≤ 0.001). Regarding the expression level of sOX40L, no statistically significant differences were observed between the two groups (*P* > 0.05) (Figures 1(e) and 1(f)).

### 3.4. Expression of mOX40 and OX40L in Patients with Different Stages of MG

**Figure 2:**
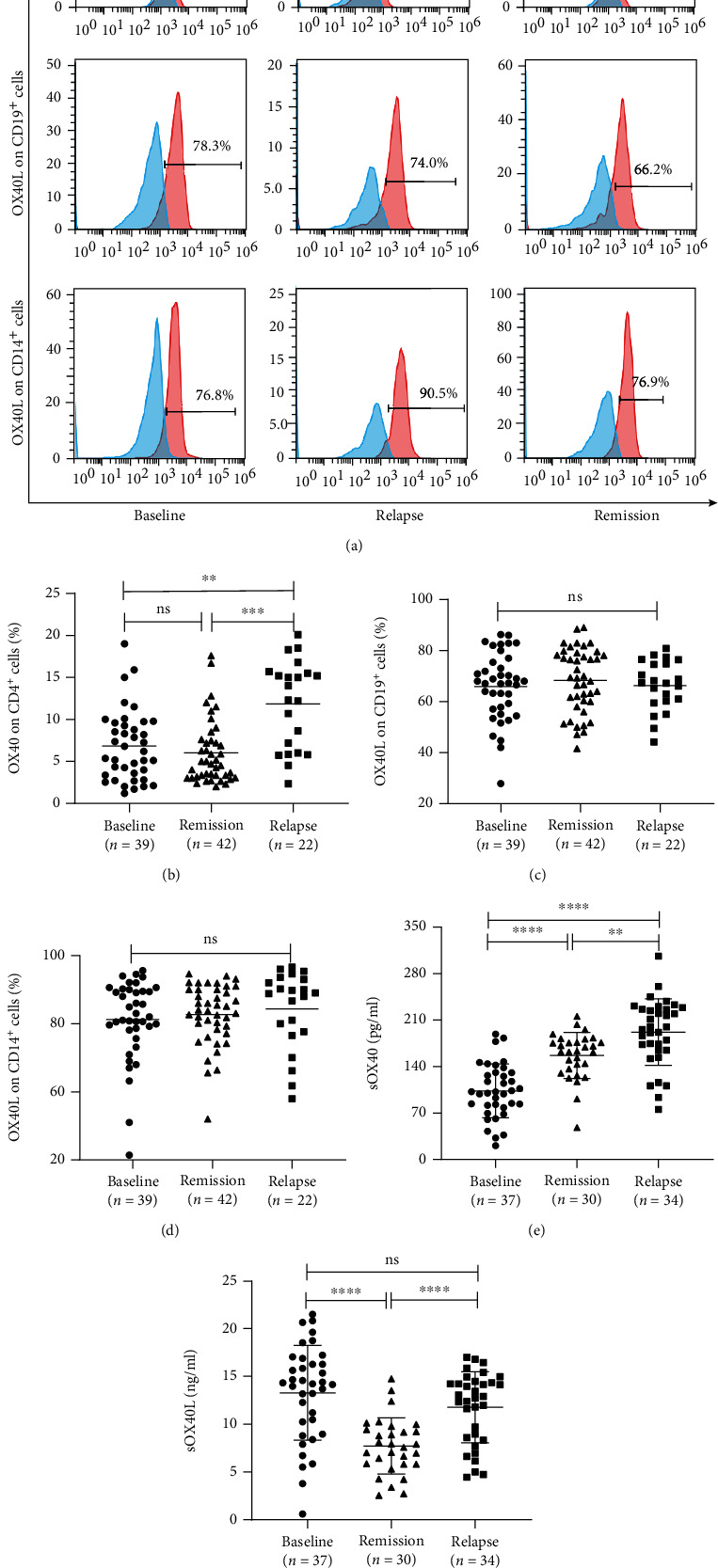
OX40/OX40L expression on the peripheral blood lymphocytes and plasma in different stages of MG patients. (a) Representative expression of OX40 and OX40L on CD4^+^ T cells, CD19^+^ B cells, and CD14^+^ mononuclear cells in MG patients at baseline, in relapse and in remission. The red lines indicate specific staining results measured by flow cytometry, and the blue lines indicate isotype controls. (b) Comparison of OX40 expression on CD4^+^ T cells in MG patients at baseline, in remission, and in relapse. (c) Comparison of OX40L expression on CD19^+^ B cells in MG patients at baseline, in remission, and in relapse. (d) Comparison of OX40L expression on CD14^+^ mononuclear cells in MG patients at baseline, in remission, and in relapse. (e) Comparison of sOX40 plasma levels in MG patients at baseline, in remission, and in relapse. (f) Comparison of sOX40L plasma levels in MG patients at baseline, in remission, and in relapse.

OX40 expression on CD4^+^ T cells was significantly higher in MG patients in relapse than in MG patients at baseline and MG patients in remission (*P* = 0.016 and *P* = 0.002, respectively), whereas OX40 expression on CD4^+^ T cells was not significantly different between MG patients at baseline and MG patients in remission (*P* > 0.05) (Table 3, Figures 2(a) and 2(b)). In addition, the expression of OX40L on CD19^+^ B cells and CD14^+^ monocytes did not significantly differ between patients with MG at baseline, in remission, and in relapse (*P* > 0.05) (Table 3, Figures 2(a), 2(c), and 2(d)).

### 3.5. Plasma Concentrations of sOX40 and sOX40L in Patients with Different Stages of MG

Compared with those in the patients with MG at baseline and in remission, the concentrations of sOX40 in the patients with MG in relapse were significantly increased (*P* ≤ 0.001 and *P* = 0.004, respectively). In addition, the expression level of sOX40 was significantly elevated in MG patients in remission compared with MG patients at baseline (*P* ≤ 0.001) (Table 3, Figure 2(e)). The sOX40L expression levels were significantly lower in MG patients in remission than in MG patients at baseline and in relapse (*P* ≤ 0.001 and *P* ≤ 0.001, respectively). Nevertheless, there was no significant difference in the sOX40L expression levels between MG patients at baseline and in relapse (*P* > 0.05) (Table 3, Figure 2(f)).

### 3.6. Effects of Immunosuppressive Drugs on the Levels of sOX40 and sOX40L in the Plasma of MG Patients

**Figure 3:**
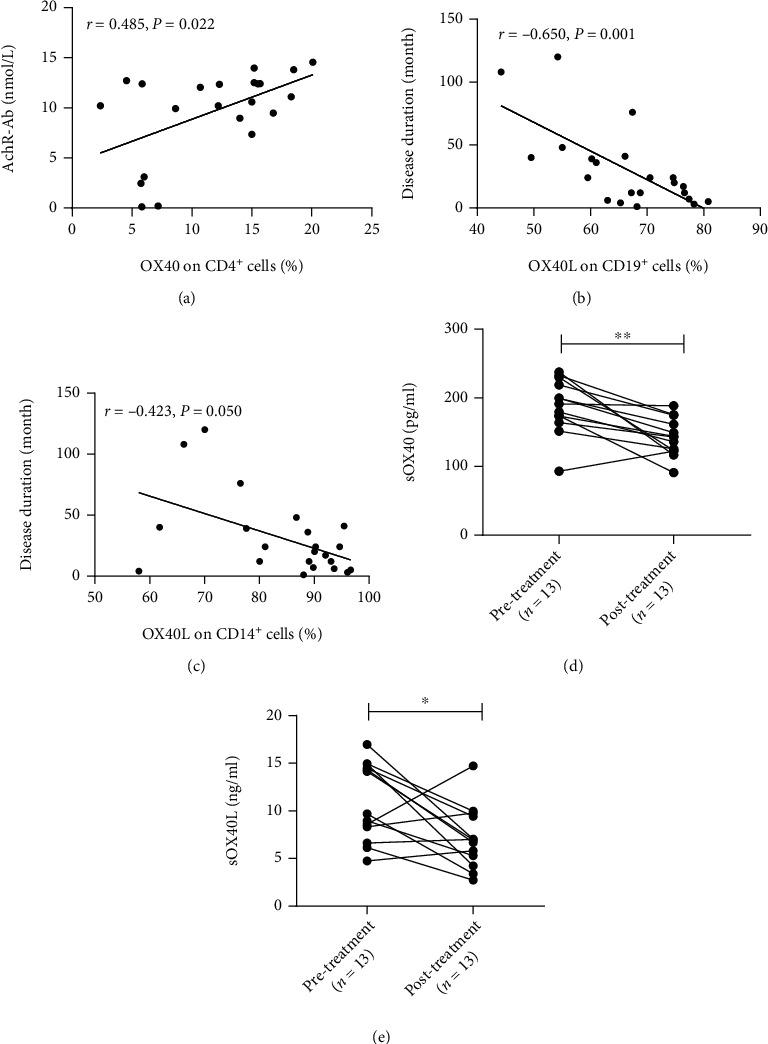
Clinical correlation diagram in patients with MG. (a) Linear correlations between the concentration of AchR-Ab and the expression of OX40 on CD4^+^ T cells in MG patients in relapse. (b) Linear correlations between the disease duration and the expression of OX40L on CD19^+^ T cells in MG patients in relapse. (c) Linear correlations between the disease duration and the expression of OX40L on CD14^+^ mononuclear cells in MG patients in relapse. (d) Comparison of sOX40 expression levels in plasma before and after immunosuppressive therapy in 13 MG patients with relapse. (e) Comparison of sOX40L expression levels in plasma before and after immunosuppressive therapy in 13 MG patients with relapse.

Plasma was retrospectively collected from 13 patients with relapsed MG and those in remission after treatment with immunosuppressive drugs. All 13 MG patients were treated with intravenous methylprednisolone (1-1.5 mg/kg/d), and four of them also received tacrolimus (0.05-0.1 mg/kg/d). ELISA results showed that the plasma levels of sOX40 and sOX40L were significantly decreased in 13 MG patients after immunosuppressive therapy (*P* = 0.002 and *P* = 0.016, respectively) (Table 4, Figures 3(d) and 3(e)).

### 3.7. Correlation with Clinical Indicators

We analyzed the correlation between laboratory parameters and clinical data (including age, QMGs, AchR-Ab concentration, and disease duration) in MG patients at different stages to explore the clinical significance of the expression of OX40 and OX40L (Table 5). The correlation analysis results demonstrated that in relapsed MG patients, the expression of OX40 on CD4^+^ T cells was positively correlated with the concentrations of AchR-Ab (*r* = 0.485, *P* = 0.022) (Figure 3(a)), while there was no significant correlation with other clinical indices (*P* > 0.05). In contrast, OX40L expression on CD19^+^ B cells and CD14^+^ monocytes was negatively correlated with disease duration (*r* = −0.650, *P* = 0.001 and *r* = −0.423, *P* = 0.050, respectively) (Figures 3(b) and 3(c)), whereas there was no significant correlation with other clinical indices (*P* > 0.05); additionally, the expression levels of sOX40 and sOX40L did not correlate significantly with clinical data (*P* > 0.05). However, there was no significant correlation between OX40 and OX40L expression and clinical parameters in MG patients at baseline and in remission (*P* > 0.05).

### 3.8. Patients with High Expression of OX40 on CD4^+^ T Cells and sOX40L Have an Increased Risk of Relapse

Patients with MG were divided into relapse and remission groups according to their prognosis. The clinical characteristics of the two groups of patients with MG are shown in Table 6. For membrane molecules, statistical analysis indicated significant differences between the two groups in terms of MGFA classification, AchR-Ab positivity rate, and QMGs (*P* < 0.05), while there were no differences in age, age of onset, sex, proportion of patients with thymoma, or treatment (*P* > 0.05). For soluble molecules, there were some differences between the two groups with respect to MGFA classification, the proportion of patients with thymoma, QMGs, and the proportion of patients treated with tacrolimus (*P* < 0.05), while there were no statistically significant differences in terms of age, age of onset, sex, AchR-Ab positivity rate, and proportion of patients treated with drugs other than tacrolimus (*P* > 0.05).

We used binary logistic regression analysis to identify factors associated with relapse in patients with MG. For membrane molecules, Table 7 indicates the results of univariable logistic regression analyses, which were used to determine the association between all the variables and relapse in patients with MG. The results suggested that the expression of OX40 on CD4^+^ T cells, MGFA classification, presence of thymoma, and AchR-Ab titers were significantly associated with relapse (*P* < 0.1). When multivariable adjustment for potential confounders was carried out, the results indicated that only the expression of OX40 on CD4^+^ T cells (adjusted OR, 1.224; 95% CI, 1.048-1.429; *P* = 0.011) and MGFA classification (adjusted OR, 7.795; 95% CI, 1.629-37.296; *P* = 0.010) were positively associated with relapse. An increase in the expression level of OX40 on CD4^+^ T cells by 1% increased the risk of relapse by 22.4%. The risk of relapse was 7.795 times higher in GMG patients than in OMG patients.

For soluble molecules, the results of the univariable regression analyses in this study showed that the expression levels of sOX40 and sOX40L, MGFA classification, presence of thymoma, concentrations of AchR-Ab, and treatment in patients with MG were significantly related to relapse (*P* < 0.1) (Table 8). After the factors that might potentially affect relapse were adjusted, our results showed that the expression level of sOX40L (adjusted OR, 1.344; 95% CI, 1.011-1.787; *P* = 0.042), MGFA classification (adjusted OR, 8.743; 95% CI, 1.185-64.488; *P* = 0.033), and treatment (adjusted OR, 0.166; 95% CI, 0.030-0.928; *P* = 0.041) were associated with relapse of MG in the study. Each point increase in sOX40L was associated with a 0.344-fold increase in the risk of relapse. GMG patients had an 8.743 times higher risk of relapse than OMG patients. Patients in the immunosuppressant-treated group had a 0.166 times higher risk of recurrence than those in the group treated with glucocorticoid alone.

## 4. Discussion

OX40, a member of the TNFR superfamily, is mainly expressed on activated CD4^+^ T cells. Its cognate ligand OX40L, a member of the TNF superfamily, is predominantly expressed on activated ADCs and on some endothelial cells, mast T cells, and activated T cells. Accumulating evidence has shown that the OX40/OX40L pathway plays a crucial role in the pathogenesis of multiple autoimmune diseases [24–27]. Furthermore, a correlation between the expression of OX40 on CD4^+^ T cells and disease severity has been observed in individuals with autoimmune diseases, such as SLE [13, 28]. The interaction between OX40 and OX40L mainly regulates the downstream PI3K-PKB-, NF-*κ*B-, and NFAT-mediated signaling pathways, regulates T cell division, promotes cytokine gene transcription and cytokine receptor expression, promotes B cell differentiation into plasma cells that produce antibodies, and inhibits apoptosis [7, 29]. Although previous studies have shown increased expression of OX40 on CD4^+^ T cells in thymomas from MG patients, little is known about the clinical significance and expression patterns of OX40 and OX40L in the peripheral blood of MG patients. Here, for the first time, we systematically investigated the expression of membrane-bound and soluble OX40 and OX40L in the peripheral blood of MG patients at different stages, as well as the effect of immunosuppressive agents (both glucocorticoids and immunosuppressants) on sOX40 and sOX40L levels.

Our results indicated that the expression of OX40 on CD4^+^ T cells was significantly higher in MG patients at baseline than in HC, which was consistent with a previous investigation [30]. In addition, our study demonstrated that OX40L expression on CD19^+^ B cells and CD14^+^ monocytes was significantly upregulated in the MG group at baseline compared to the HC group. In addition to their membrane-bound forms, costimulatory molecules were also present in their soluble forms. We measured the expression of sOX40 and sOX40L in the peripheral blood of MG patients and found that the expression of sOX40 was significantly lower in MG patients at baseline than in HC, while plasma sOX40L levels were not significantly different between the two groups. Soluble costimulatory molecules are generated through the proteolytic cleavage [31] or mRNA splicing [32] of membrane-bound molecules. It was suggested that sOX40 can bind to OX40L on APC cells, thus interfering with positive signal transmission from OX40L to OX40^+^ T cells and inhibiting T cell activation [33–35]. We hypothesized that the decrease in sOX40 levels in patients with MG may be related to a decrease in mOX40 shedding and that the reduced sOX40 levels may result in reduced binding of sOX40 to membranous and soluble OX40L, thereby increasing the opportunity for OX40L to bind to membranous OX40, leading to a relative increase in positive signaling to CD4^+^ T cells and relative immune hyperfunction.

MG is an intricate and heterogeneous disease, so we investigated, for the first time, the dynamics of membrane-bound and soluble OX40 and OX40L during the disease onset and progression. For membrane molecules, the expression of OX40 on CD4^+^ T cells was significantly increased in patients with relapsed MG compared with patients with MG at baseline and in remission. However, there was no significant difference in the expression of OX40L on CD19^+^ B cells and CD14+ monocytes in patients with MG at baseline, in relapse, and in remission. These phenomena suggested that the OX40/OX40L signaling pathway may function mainly in the later stage of MG and may be associated with disease activity. We presumed that this may be related to the mechanism by which the OX40/OX40L signaling pathway functions in T cells. On the one hand, from the perspective of T cell survival, Song et al. [36] found that OX40-deficient T cells normally differentiate and proliferate into effector T cells 2-3 days after the activation of TCR signaling. Nevertheless, the survival rate was significantly decreased after 12-13 days of activation, suggesting that OX40 signaling is not essential in the early stage of T cell activation but mainly promotes T cell activation and maintains T cell survival in the later stage. On the other hand, from the perspective of T cell function, OX40-OX40L interactions promote the generation of memory T cells and maintain their survival [33–35, 37], and the long-term survival of memory CD4^+^ T cells after antigen restimulation allows their rapid differentiation into effector T cells, which may be one of the causes of disease recurrence [38].

For soluble molecules, similar to the expression level of mOX40, the expression level of sOX40 was significantly higher in MG patients in relapse than in baseline and remission, which may be related to the corresponding increase in the levels of sOX40 due to mOX40 shedding. Unlike the expression level of mOX40, the expression level of sOX40 was significantly higher in MG patients in remission than in those at baseline. Dynamic observation of the molecules showed a significant decrease in the sOX40L levels in patients with MG in remission compared to those in patients with MG at baseline and relapsed MG. It was experimentally demonstrated that sOX40L promotes the late proliferation and activation of T cells but does not affect early proliferation and activation [39, 40], and sOX40L can bind to OX40 on activated T cells and allow the T cells to receive sustained positive stimulation signals [41, 42]. The concentrations of sOX40L were dozens of times higher than those of sOX40, and the expression of OX40L on CD19^+^ B cells and CD14^+^ monocytes was negatively correlated with disease duration in patients with relapsing MG. We hypothesized that sOX40L may be a functional molecule that acts in the later stage of MG, binding to OX40 on activated T cells and enhancing T cell activation. This may be an important mechanism for initiating the reimmune response, but further experimental evidence is needed to confirm this.

In addition, we also examined the expression levels of sOX40 and sOX40L in plasma before and after immunosuppressive treatment in 13 patients with relapsing MG, and the results showed that the plasma levels of sOX40 and sOX40L in patients with MG in remission who have received immunosuppressive treatment were significantly decreased compared with those in patients who had not yet received treatment. These findings suggest that the expression of soluble molecules may be influenced by immunosuppressive agents. The study found that the serum OX40L level was decreased in asthmatic patients after inhaled corticosteroid treatment [43]. Therefore, we hypothesized that immunosuppressive agents, such as corticosteroids, may inhibit OX40/OX40L signaling by inhibiting the expression of sOX40 and sOX40L, leading to immunosuppressive effects.

Correlation analysis indicated that in patients with relapsed MG, the expression of OX40 on CD4^+^ T cells was positively correlated with the concentrations of AchR-Ab. For membrane molecules, binary regression analysis showed that the expression of OX40 on CD4^+^ T cells and MGFA classification were associated with relapse of MG. For soluble molecules, the expression levels of sOX40L, MGFA classification, and treatment correlated with relapse in MG patients. Thus, CD4^+^ OX40 and sOX40L may be expected to be biomarkers for monitoring relapse in MG patients after immunosuppressive therapy, and further studies in large cohorts are required in the future. Additionally, patients with GMG are more likely to relapse, suggesting that the disease severity of MG may be associated with relapse. However, MG patients treated with glucocorticoids alone were more likely to relapse than those treated with immunosuppressants. Therefore, for patients with an initial diagnosis of MG, intense immunotherapy should be aggressively administered at the beginning of treatment to rapidly control the disease and thus reduce the risk of disease relapse after treatment.

The primary advantage of our study is that we first dynamically investigated the changes in the levels of membrane-bound and soluble OX40 and OX40L in the peripheral blood of patients with MG at different stages. Other strengths include our first study of the effects of immunosuppression and disease status on sOX40 and sOX40L levels in the peripheral blood of MG patients. Our research also had some limitations. First, the patients with MG included in this study were nonconsecutively enrolled from a single center, which may result in selection bias. The sample size of patients with MG in each phase was relatively small, and the insufficient sample size may reduce the statistical effect of the conclusions. Second, our study did not elucidate the specific mechanisms by which the OX40/OX40L signaling pathway participates in the immunopathological process of MG. Third, the nature of the research was observational. We could not demonstrate a causal relationship between the expression level of OX40 on CD4^+^ T cells and the relapse of MG. Fourth, this study is a cross-sectional study rather than a longitudinal study, and the subjects included in the membrane-bound molecule cohort and the soluble molecule cohort were not identical, which may affect the accuracy of the conclusions. Future large longitudinal cohort studies should address whether these findings are contingent and modifiable. Fifth, this study measured only the concentrations of AchR-Ab in all patients with MG but did not measure other myasthenia gravis-related antibodies, such as anti-MuSK antibodies, anti-LRP4 antibodies, anti-titin, and anti-RyR antibodies, in all the patients.

## 5. Conclusions

This study showed that OX40 and OX40L are abnormally expressed in the peripheral blood of patients with MG. OX40 on CD4^+^ T cells may be associated with disease activity. sOX40 and sOX40 levels may be related to both disease status and immunosuppressive agent administration. The OX40/OX40L pathway may be involved in the immunopathological process of MG and may function primarily in the later stage of MG. The regulation of the OX40/OX40L pathway may provide new targets and directions for the treatment of MG.

## Figures and Tables

**Table 1:** The clinical characteristics of the study population.

Clinical features	Membrane-bound molecules					Soluble molecules				
	Baseline (*n* = 39)	Remission (*n* = 42)	Relapse (*n* = 22)	HC (*n* = 36)	*P* value	Baseline (*n* = 37)	Remission (*n* = 30)	Relapse (*n* = 34)	HC (*n* = 30)	*P* value
Age (years)	49.0 (37.0, 63.0)	46.0 (36.0, 58.0)	48.0 (32.0, 58.0)	47.0 (32.5, 58.5)	0.754	43.0 (33.0, 66.0)	39.0 (34.0, 57.0)	50.5 (40.0, 62.0)	44.5 (34.0, 54.0)	0.258
Age of onset, *n* (%)										
EOMG (age < 50 y)	13 (33.3)	17 (40.4)	12 (54.6)	n.a.	0.269	16 (43.2)	19 (63.3)	16 (47.1)	n.a.	0.233
LOMG (age ≥ 50 y)	9 (23.1)	18 (42.9)	8 (36.4)	n.a.	0.165	16 (43.2)	11 (36.7)	18 (52.9)	n.a.	0.417
Sex, *n* (%)					0.557					0.141
Female	20 (51.3)	27 (64.3)	14 (63.6)	19 (52.8)		19 (51.3)	21 (70.0)	20 (58.8)	23 (76.7)	
Male	19 (49.7)	15 (35.7)	8 (36.4)	17 (47.2)		18 (48.7)	9 (30.0)	14 (41.2)	7 (23.3)	
MGFA classification, *n* (%)					0.001					≤0.001
OMG	25 (64.1)	30 (71.4)	3 (13.6)	n.a.		22 (59.5)	19 (63.3)	3 (8.8)	n.a.	
GMG	14 (35.9)	12 (28.6)	19 (86.4)	n.a.		15 (40.5)	11 (36.7)	31 (91.2)	n.a.	
Thymoma, *n* (%)					0.061					0.005
Without	32 (82.1)	35 (83.3)	13 (59.1)	n.a.		27 (73.0)	21 (70.0)	13 (38.2)	n.a.	
With	7 (17.9)	7 (16.7)	9 (40.9)	n.a.		10 (27.0)	9 (30.0)	21 (61.8)	n.a.	
AchR-Ab, *n* (%)					0.003					1.000
Positive, *n* (%)	22 (56.4)	35 (83.3)	20 (90.9)	n.a.		32 (86.5)	30 (100.0)	34 (100.0)	n.a.	
Negative, *n* (%)	19 (43.6)	7 (16.7)	2 (9.1)	n.a.		5 (13.5)	0 (0.0)	0 (0.0)	n.a.	
Disease duration (months)	3 (1.0, 24.0)	22.5 (8.0, 46.0)	22 (7.0, 40.0)	n.a.	≤0.001	1 (1.0, 2.0)	15 (10.0, 38.0)	20 (3.0, 52.0)	n.a.	≤0.001
QMGs	7.0 (5.0, 10.5)	7.0 (4.0, 8.0)	13.0 (11.0, 18.0)	n.a.	≤0.001	8.0 (6.0, 12.0)	7.0 (6.0, 9.0)	14.0 (12.0, 20.0)	n.a.	≤0.001

Abbreviations: HC: healthy control; EOMG: early-onset myasthenia gravis; LOMG: late-onset myasthenia gravis; MGFA: Myasthenia Gravis Foundation of America; AchR-Ab: acetylcholine receptor antibodies; QMGs: quantitative myasthenia gravis scores.

**Table 2:** OX40 and OX40L expression in patients with MG at baseline and healthy controls.

Group	Membrane-bound molecules				Soluble molecules		
	No.	CD4^+^OX40^+^ (%)	CD19^+^OX40L^+^ (%)	CD14^+^OX40L^+^ (%)	No.	sOX40 (pg/ml)	sOX40L (ng/ml)
MG at baseline	39	6.20 (3.48, 9.43)	67.30 (57.45, 74.25)	82.00 (78.35, 89.75)	37	103.29 ± 40.54	13.27 ± 4.95
HC	36	2.27 (1.32, 3.95)	58.15 (50.70, 63.70)	78.25 (71.80, 85.50)	30	169.24 ± 41.81	12.58 ± 4.83
Test of value		-4.311	-2.535	-2.222		-6.529	0.576
*P* value		≤0.001	0.011	0.026		≤0.001	0.566

**Table 3:** OX40 and OX40L expression in patients with MG at different stages.

Group	Membrane-bound molecules				Soluble molecules		
	No.	CD4^+^OX40 (%)	CD19^+^OX40L^+^ (%)	CD14^+^OX40L^+^ (%)	No.	sOX40 (pg/ml)	sOX40L (ng/ml)
Baseline	39	6.20 (3.48, 9.43)^a,b^	67.30 (57.45, 74.25)^a,b^	82.00 (78.35, 89.75)^a,b^	37	103.29 ± 40.54^a,b^	13.27 ± 4.95^a.b^
Remission	42	4.85 (3.17, 7.47)^c^	69.35 (60.00, 78.20)^c^	83.85 (78.30, 90.00)^c^	30	156.62 ± 34.56^c^	7.73 ± 2.94^c^
Relapse	22	13.15 (6.00, 15.50)	67.30 (60.20, 74.80)	88.90 (77.60, 93.00)	34	191.88 ± 50.04	11.77 ± 3.71
a		*Z* = 0.626, *P* = 1.000	*Z* = −0.618, *P* = 1.000	*Z* = −0.343, *P* = 1.000		*P* ≤ 0.001	*P* ≤ 0.001
b		*Z* = −2.995, *P* = 0.016	*Z* = 0.099, *P* = 1.000	*Z* = −1.251, *P* = 1.000		*P* ≤ 0.001	*P* = 0.355
c		*Z* = 3.563, *P* = 0.002	*Z* = −0.622, *P* = 1.000	*Z* = 0.977, *P* = 1.000		*P* = 0.004	*P* ≤ 0.001

a: baseline vs. remission; b: baseline vs. relapse; c: remission vs. relapse.

**Table 4:** Comparison of sOX40 and sOX40L expression in the plasma of patients with relapsing MG before and after immunosuppressive treatment.

Stage	sOX40 (pg/ml)	sOX40L (ng/ml)	QMGs
Before treatment (*n* = 13)	188.49 ± 39.66	10.99 ± 4.09	14.92 ± 5.25
Remission after treatment (*n* = 13)	142.83 ± 27.49	7.16 ± 3.23	8.62 ± 2.79
Statistic of test	4.057	2.818	4.879
*P* value	0.002	0.016	≤0.001

**Table 5:** Correlation between clinical data and laboratory parameters.

Stage	Clinical characteristics		OX40 on CD4^+^ cells (%)	OX40L on CD19^+^ cells (%)	OX40L on CD14^+^ cells (%)	sOX40 (ng/ml)	sOX40L (ng/ml)
	Age (years)	*r*	-0.018	0.074	0.063	0.090	-0.156
		*P*	0.915	0.654	0.702	0.596	0.357
		*n*	39	39	39	37	37
	QMGs	*r*	0.032	-0.031	0.036	0.126	-0.119
		*P*	0.846	0.851	0.829	0.459	0.483
		*n*	39	39	39	37	37
Baseline	AchR-Ab (nmol/L)	*r*	0.152	-0.024	0.015	-0.178	-0.241
		*P*	0.354	0.884	0.928	0.292	0.150
		*n*	39	39	39	37	37
	Disease duration (months)	*r*	-0.215	-0.258	-0.035	-0.050	-0.032
		*P*	0.188	0.127	0.831	0.770	0.849
		*n*	39	39	39	37	37
	Age (years)	*r*	-0.095	-0.091	0.066	0.110	-0.153
		*P*	0.552	0.569	0.679	0.563	0.419
		*n*	42	42	42	30	30
	QMGs	*r*	0.156	-0.008	-0.148	0.104	0.255
		*P*	0.324	0.962	0.350	0.583	0.173
		*n*	42	42	42	30	30
Remission	AchR-AR (nmol/L)	*r*	-0.008	-0.008	-0.050	-0.181	-0.049
		*P*	0.964	0.959	0.771	0.339	0.799
		*n*	37		37	30	30
	Disease duration (months)	*r*	0.112	-0.008	-0.107	-0.004	-0.095
		*P*	0.482	0.959	0.498	0.983	0.618
		*n*	42	42	42	30	30
	Age (years)	*r*	-0.356	-0.316	-0.339	0.151	0.101
		*P*	0.103	0.152	0.123	0.394	0.954
		*n*	22	22	22	34	34
	QMGs	*r*	0.213	0.155	0.281	0.070	-0.129
		*P*	0.342	0.491	0.205	0.693	0.467
		*n*	22	22	22	34	34
Relapse	AchR-AR (nmol/L)	*r*	0.485	0.102	0.226	0.048	-0.051
		*P*	0.022	0.653	0.311	0.790	0.772
		*n*	22	22	22	34	34
	Disease duration (months)	*r*	-0.001	-0.650	-0.423	0.204	0.224
		*P*	0.998	0.001	0.050	0.248	0.202
		*n*	22	22	22	34	34

**Table 6:** Clinical characteristics of patients with MG in relapse and remission.

Clinical characteristics	Membrane-bound molecules			Soluble molecules		
	Remission (*n* = 42)	Relapse (*n* = 22)	*P* value	Remission (*n* = 30)	Relapse (*n* = 34)	*P* value
Age (years)	46.0 (36.0, 58.0)	48.0 (32.0, 58.0)	0.754	39.0 (34.0, 57.0)	50.5 (40.0, 62.0)	0.258
Age of onset, *n* (%)						
EOMG (age < 50 y)	17 (40.4)	12 (54.6)	0.283	19 (63.3)	16 (47.1)	0.192
LOMG (age ≥ 50 y)	18 (42.9)	8 (36.4)	0.192	11 (36.7)	18 (52.9)	
Sex, *n* (%)			0.557			0.141
Female	27 (64.3)	14 (63.6)		21 (70.0)	20 (58.8)	
Male	15 (35.7)	8 (36.4)		9 (30.0)	14 (41.2)	
MGFA classification, *n* (%)			≤0.001			≤0.001
OMG	30 (71.4)	3 (13.6)		19 (63.3)	3 (8.8)	
GMG	12 (28.6)	19 (86.4)		11 (36.7)	31 (91.2)	
Thymoma, *n* (%)			0.050			0.011
Without	35 (83.3)	13 (59.1)		21 (70.0)	13 (38.2)	
With	7 (16.7)	9 (40.9)		9 (30.0)	21 (61.8)	
AchR-Ab, *n* (%)			0.003			1.000
Positive, *n* (%)	35 (83.3)	20 (90.9)		30 (100.0)	34 (100.0)	
Negative, *n* (%)	7 (16.7)	2 (9.1)		0 (0.0)	0 (0.0)	
QMGs	7.0 (4.0, 8.0)	13.0 (11.0, 18.0)	≤0.001	7.0 (6.0, 9.0)	14.0 (12.0, 20.0)	≤0.001
Treatment, *n* (%)						
Pyridostigmine	2 (4.8)	3 (13.6)	0.329	0 (0)	0 (0)	/
Glucocorticoid	18 (42.9)	10 (45.5)	1.000	15 (50)	26 (76.5)	0.218
Immunosuppressants						
Azathioprine	8 (19.0)	1 (4.5)	0.147	4 (13.3)	3 (8.8)	0.679
Tacrolimus	11 (26.2)	5 (22.7)	1.000	9 (30)	2 (5.9)	0.018
Mycophenolate mofetil	1 (2.4)	2 (9.1)	0.270	0 (0)	1 (2.9)	1.000
Cyclophosphamide	2 (4.8)	1 (4.5)	1.000	2 (6.7)	2 (5.9)	1.000

Abbreviations: HC: healthy control; EOMG: early-onset myasthenia gravis; LOMG: late-onset myasthenia gravis; MGFA: Myasthenia Gravis Foundation of America; AchR-Ab: acetylcholine receptor antibodies; QMGs: quantitative myasthenia gravis scores.

**Table 7:** Univariate and multivariate logistic regression risk models of the risk of relapse of MG (membrane-bound molecules).

Variables	Univariate analysis		Multivariate analysis	
	HR (95% CI)	*P* value	HR (95% CI)	*P* value
CD4^+^OX40 (%)	1.289 (1.132-1.469)	≤0.001	1.224 (1.048-1.429)	0.011
CD19^+^OX40L (%)	0.985 (0.942-1.030)	0.518		
CD14^+^OX40L (%)	1.017 (0.963-1.075)	0.540		
Sex, *n* (%)				
Male	Ref			
Female	0.972 (0.332-2.845)	0.959		
Age (years)	1.011 (0.980-1.044)	0.479		
Follow-up (months)	0.994 (0.982-1.006)	0.302		
MGFA classification				
OMG	Ref			
GMG	15.833 (3.945-63.540)	≤0.001	7.795 (1.629-37.296)	0.010
Thymoma				
Without	Ref			
With	5.000 (1.560-16.028)	0.007	1.268 (0.237-6.799)	0.782
AchR-Ab (nmol/L)	1.193 (1.052-1.353)	0.006	1.064 (0.920-1.230)	0.405
Treatment				
Pyridostigmine	Ref			
Glucocorticoid	3.667 (0.522-25.773)	0.192		
Immunosuppressant	1.358 (0.454-4.059)	0.584		

Abbreviations: OMG: ocular myasthenia gravis; GMG: generalized myasthenia gravis; MGFA: Myasthenia Gravis Foundation of America; AchR-Ab: acetylcholine receptor antibodies.

**Table 8:** Univariate and multivariate logistic regression risk models of the risk of relapse of MG (soluble molecules).

Variables	Univariate analysis		Multivariate analysis	
	HR (95% CI)	*P* value	HR (95% CI)	*P* value
sOX40 (pg/ml)	1.018 (1.005-1.031)	0.008	1.008 (0.981-1.037)	0.562
sOX40L (ng/ml)	1.391 (1.171-1.652)	≤0.001	1.344 (1.011-1.787)	0.042
Sex, *n* (%)				
Male	Ref			
Female	0.673 (0.241-1.884)	0.451		
Age	1.023 (0.991-1.056)	0.168		
Follow-up (month)	1.001 (0.995-1.008)	0.681		
MGFA classification				
OMG	Ref			
GMG	19.000 (4.705-76.727)	≤0.001	8.743 (1.185-64.488)	0.033
Thymoma				
Without	Ref			
With	3.267 (1.173-9.096)	0.023	4.808 (0.678-34.120)	0.116
AchR-Ab (nmol/L)	1.247 (1.098-1.415)	0.001	1.134 (0.920-1.398)	0.240
Treatment				
Glucocorticoid	Ref			
Immunosuppressant	3.500 (1.209-10.131)	0.021	0.166 (0.030-0.928)	0.041

Abbreviations: OMG: ocular myasthenia gravis; GMG: generalized myasthenia gravis; MGFA: Myasthenia Gravis Foundation of America; AchR-Ab: acetylcholine receptor antibodies.

## Data Availability

The authors will provide all raw data supporting the conclusions of this manuscript without any reservation.

## Abbreviations

MG: Myasthenia gravis
HC: Healthy control
PB: Peripheral blood
mOX40: Membrane-bound OX40
mOX40L: Membrane-bound OX40L
sOX40: Soluble OX40
sOX40L: Soluble OX40L
ELISA: Enzyme-linked immunosorbent assay
OMG: Ocular myasthenia gravis
GMG: Generalized myasthenia gravis
QMGs: Quantitative myasthenia gravis scores
AchR-Ab: Acetylcholine receptor antibodies
MHC: Major histocompatibility complex
TNFR: Tumor necrosis factor receptor
TNF: Tumor necrosis factor
ADCs: Antigen-presenting cells
DCs: Dendritic cells
MS: Multiple sclerosis
SLE: Systemic lupus erythematosus
RA: Rheumatoid arthritis
MGFA: Myasthenia Gravis Foundation of America
CI: Confidence interval.
